# Trace element mobility during the injection of water-dissolved CO_2_ into basalts

**DOI:** 10.1038/s41598-026-57307-6

**Published:** 2026-06-10

**Authors:** Abdirizak Omar, Mouadh Addassi, Niccolo Menegoni, Serguey Arkadakskiy, Jakub Fedorik, Zeyad Ahmed, Noushad Kunnummal, Thomas Finkbeiner, Sigurdur R. Gislason, Abdulkader Afifi, Hussein Hoteit, Eric H. Oelkers

**Affiliations:** 1 Physical Science and Engineering Division, King Abdullah University of Science and Technology, Thuwal, Saudi Arabia; 2 Saudi Aramco, Dhahran, Saudi Arabia; 3 University of Pavia, Pavia, Italy; 4 University of Iceland, Reykjavik, Iceland

**Keywords:** CCS, CO_2_ mineralization, Basalt, Trace element mobility, Groundwater quality, Jizan pilot, Biogeochemistry, Environmental sciences, Solid Earth sciences

## Abstract

In-situ carbon mineralization in basalts has been identified as a permanent and scalable method to sequester CO_2_ in the subsurface. The extent of potentially toxic trace element mobilization during basalt dissolution, however, remains poorly constrained. The Jizan carbon sequestration pilot was undertaken to assess the potential for carbon disposal through the mineralization of water-dissolved CO_2_ injected into subsurface basalts in arid regions. The temporal evolution of trace element and potentially toxic metal concentrations in the Jizan groundwater during and following the injection of CO_2_-charged water and its recirculation has been assessed. Twenty-eight representative production well fluid samples were selected and analyzed by ICP-MS. Element mobility was quantified by comparing the increase in concentration of the selected elements in the aqueous phase to the corresponding increase in sodium concentration. Results indicate that the mobility of trace elements in the Jizan system is limited, with the average mobility of most trace elements remaining below 20% of that of sodium. Furthermore, the maximum measured concentrations of most trace elements are significantly below the acceptable levels in drinking water before, during, and after the CO_2_ injection. These observations suggest the risk of groundwater pollution during in-situ carbon mineralization efforts in basalt is likely minimal.

**Supplementary Information:**

The online version contains supplementary material available at 10.1038/s41598-026-57307-6.

## Introduction

This study focuses on the behavior of trace and potentially toxic elements during and after CO_2_ injection in the Jizan in-situ CO_2_ mineralization pilot^1^. This pilot demonstrated that the majority of water-dissolved CO_2_ was mineralized by its reaction with subsurface basalts in less than a year. In-situ mineralization of CO_2_ injected into subsurface basalts, as demonstrated in the Jizan pilot, is reported to be a safe and permanent carbon disposal method^2–10^. This carbon storage process was first demonstrated as part of the CarbFix projects^11,12^. The dissolution of basalts in the presence of acidic aqueous CO_2_ promotes carbon disposal in two ways. First, the dissolution of basalts releases the divalent cations required for forming carbonate minerals. Second, basalt dissolution increases the fluid pH, favoring carbonate mineral precipitation^13^. Carbonate minerals provide a long-term storage host for disposed carbon, as these minerals are stable over geologic time scales^12,14^. The abundance of basaltic reservoirs globally suggests that this carbon sequestration approach could be significantly upscaled in the future^15^. Notably, subsurface basaltic reservoirs provide CO_2_ sinks for regions that lack other subsurface CO_2_ sequestration options, such as saline aquifers and depleted hydrocarbon reservoirs^16^.

The injection of acidic CO_2_-charged water and the subsequent dissolution of minerals in a basalt could, however, lead to trace element mobilization^17–23^. The risk of toxic metal mobility in response to CO_2 _disposal efforts has motivated several past studies. Clark et al.^17^ reported concentrations of trace and toxic elements from the CarbFix 1 and 2 pilots in Iceland. Their study showed that measured concentrations of these elements were significantly lower than those calculated from stoichiometric basalt dissolution, suggesting their coprecipitation with secondary minerals, and thus a low risk of groundwater contamination. McGrail et al.^24^ reported slightly increased concentrations of Fe, Mn, Sr, and B related to reactions between CO_2_ and the ferromagnesian minerals in subsurface basalts during the Wallula pilot project. Islam^25^ performed laboratory experiments on basalt glass and altered basalt to assess trace element mobility due to their interaction with CO_2_. This study reported increasing aqueous Al, Fe, and Mn concentrations, leading to concentrations exceeding the 2017 World Health Organization safe drinking water guidelines levels. Karamalidis et al.^20^ performed experiments to investigate trace metal release during CO_2_-rich brine interaction with caprocks and reservoir rocks from various carbon sequestration project sites. Their results showed relatively low trace metal mobilization in basalt compared to other rock types. Flaathen et al.^18^ studied the chemical evolution of groundwaters at Mt. Hekla (Iceland) as an analogue to geoengineered basalt carbon sequestration systems. They concluded that most trace metals released by the dissolution of basalt by natural acidic CO_2_-rich waters are largely immobilized by the formation of carbonate and Fe(III) (oxy)hydroxide minerals as the groundwaters are neutralized by continued basalt dissolution. Romano et al.^23^ performed CO_2_-water-basalt batch reactor experiments and observed notable trace element mobilization at low pH but significantly less mobilization at pH > 6. Galeczka et al.^19^ quantified the relative mobility and release rate of major and trace elements during pure water and CO_2_-charged water interactions with mid-oceanic ridge basaltic glass during flow through experiments at 22 °C and 50 °C, 10^− 5.7^ to 22 bar partial pressure of CO_2_ and pH of 9.5 to 3.4. The mobility and aqueous concentration of several elements increased significantly with the addition of CO_2_ to the inlet fluids resulting in a pH of 3.4–4.5. Some metals, including Mn, Cr, Al, and As exceeded the allowable drinking water limits in the low pH solutions. The elements As and B were most mobile in all experiments and Ti the least mobile. In contrast, results from the CarbFix studies suggest that the mobility of trace and potentially toxic elements is limited during and after the subsurface mineralization of water-dissolved CO_2_ in subsurface basalts^17^.

In-situ CO_2_ mineralization has been considered in various locations globally for CO_2_ disposal^26–28^. This technology is currently being explored in the Kingdom of Saudi Arabia to achieve its net-zero emissions targets^16,29^. The presence of reactive volcanic rocks, such as basalt, provides carbon disposal opportunities in the Kingdom’s western region, which has limited sedimentary basins commonly considered for subsurface carbon disposal^30–33^. Of these, the Jizan Group, a thick sequence of Late-Oligocene to Early-Miocene mafic and felsic volcanic rocks located in southwest Saudi Arabia, may provide a large-scale carbon disposal opportunity^31,34^. The Jizan Group is estimated to have a CO_2_ sequestration capacity of up to ~ 4.2 GtCO_2_; enough to dispose of several decades of emissions from the numerous industries in its proximity^32^.

To access the potential for in-situ CO_2_ mineralization in Saudi Arabia, Saudi Aramco drilled 5 wells into the Jizan Group basalt 24 km NNE of the Jizan Economic City^1^. Two of these wells were used for injection and monitoring during an in-situ CO_2_ mineralization pilot, whereby 131 tons of CO_2_ was co-injected with water into the Jizan Group basalt^1,16,35^. As water in the Jizan region is limited, this pilot project developed a closed-loop system that continuously recirculated water from the production well to the injection well before, during, and after CO_2_ injection into the subsurface. The CO_2_ was added to the circulating fluid in quantities such that the gas dissolved completely before entering the subsurface basalts. The pilot was monitored for more than 10 months after the end of the CO_2_ injection. Several related studies describing the pilot project, characterizing the geology, fault and fracture networks, geochemistry, and mineralization potential have been published^1,16,31,32,35^.

The aim of this study is to investigate trace and potentially toxic element mobility in the Jizan reservoir water during the in-situ CO_2_ mineralization pilot project. Toward this goal, we regularly collected the fluids from the production well and measured their elemental compositions. These concentrations are compared with the corresponding compositions of basalts collected from the target subsurface injection aquifer and aqueous fluid sodium concentrations. Here, we report the temporal evolution of the measured dissolved trace and potentially toxic element concentrations and use these results to (1) quantify the mobility of these elements and (2) assess the potential fate of these elements during the injection of CO_2_-charged water into subsurface basalts.

## Methods

### Description of Jizan pilot project

**Fig. 1:**
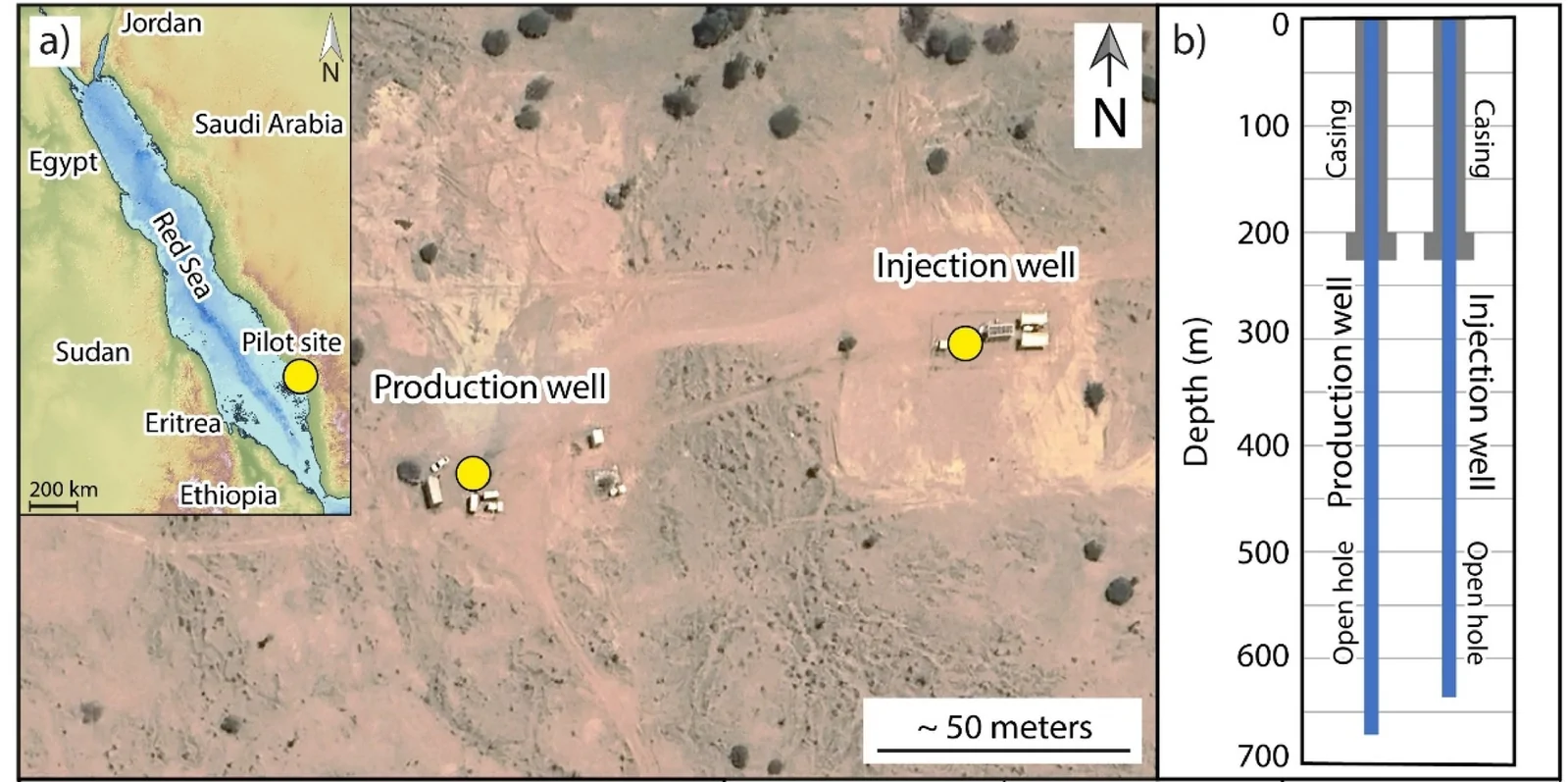
(**a**) Map showing the location of the Jizan pilot project, and an aerial image showing the relative locations of the injection and production wells. Satellite image from Google Earth (Google; includes data from Airbus). (**b**) A schematic illustration of the injection and production wells showing their casing and open-hole completion intervals.

The Jizan CO_2_ mineralization pilot site is located 24 km NNE of the Jizan Economic City, which hosts several point-source CO_2 _emitters, including refineries, power plants, and water desalination plants. This pilot has been previously described in detail by Oelkers et al.^1^. The pilot site sits above the Jizan Group basalt, with ~ 30 m of conglomerate-sandstone-shale sediments between the surface and the top of the Jizan Group. Five vertical wells ranging from 120 to 1000 m below surface (mbs) in depth were drilled into the Jizan basalts. Of these, two wells, positioned approximately 130 m apart, were designated as injection and production wells, as shown in Fig. 1. These wells were cased and cemented to approximately 250 mbs and completed as open holes below. A downhole electric submersible pump was installed in the production well to withdraw aquifer water, which was co-injected with CO_2_ in the injection well.

Beginning March 29, 2023, water was continuously pumped from the production well and reinjected through the injection well at a flow rate of 2.6 kg/s. CO_2_ was intermittently co-injected between 16 and 25 May 2023 to test the system. Continuous CO_2_ injection began on May 31, 2023. Pure CO_2_, compressed to a pressure of 14 bar, was released at a depth of 150 mbs into the downflowing water in the injection well using a sparger. The injected CO_2_ to water mass ratio was 1:65, ensuring complete dissolution of CO_2_ in the water. By July 7, 2023, when CO_2_ injection was stopped, a total of 131 tons of CO_2_ had been injected into the subsurface. Water circulation was continued, with production well fluid samples collected periodically for analyses.

### Whole rock chemical analysis

Ten representative drill-cutting samples from the production well (Fig. 1) collected from depths ranging between 40 and 660 mbs were characterized by X-ray Fluorescence (XRF) and Inductively Coupled Plasma Mass Spectrometry (ICP-MS) to determine their elemental composition. The drill cuttings were first washed using deionized water to remove impurities from the drilling and dried overnight in an oven at 45 °C. The dried cuttings were subsequently ground to a fine powder using a tungsten carbide ASC Scientific Jaw Crusher.

For the XRF analysis, approximately 1 g of the ground rock powder was mixed with 9 g of a XRF Scientific X-ray flux powder composed of 66% lithium tetraborate and 34% lithium metaborate. The resulting powder mixture was melted at 1050 °C in an Eagon 2 Fusion furnace and poured to form a homogenous pellet. This pellet was cooled and analyzed on a Bruker S8 TIGER XRF instrument. The limit of detection (LOD) of this analysis was < 150 ppm for all elements, and the uncertainty was < 1% for the major elements and < 3% for the minor elements. Loss on ignition (LOI) was measured after heating the samples to 950 °C in a muffle furnace for 12 h. The mass of the samples was measured before and after heating using a Mettler Toledo XP205 analytical balance.

For the ICP-MS analysis, the crushed and cleaned rock samples were first digested following the method of Cherviakouski et al.^36^ which is optimized for basalt samples. Ten representative samples, one blank, and 1 certified reference material (CRM – JB-3 basalt) were prepared. The polytetrafluoroethylene (PTFE) tubes and caps used for digestion were pre-cleaned by soaking them in aqueous 2% nitric acid for 1 week and rinsing with Milli-Q ultrapure deionized water (18.2 MΩ.cm). Approximately 50 mg of each sample powder was weighed on a Mettler Toledo XP205 analytical balance and placed into the PTFE tubes. One ml of VWR 70% ultrapure double-distilled nitric acid and 1.5 ml of Honeywell 48% trace-grade hydrofluoric acid were added to the tubes for digestion. The tubes were placed in a Milestone ultraCLAVE digestion system at 240 °C for up to 70 min. After digestion, 0.1 ml of Sigma-Aldrich 70% trace-grade perchloric acid was added to each PTFE tube, and the tubes were placed on a Savillex HPX-200 heating pad at 130 °C for 5 h. After evaporation of excess hydrofluoric acid, 2 ml of 70% ultrapure double-distilled nitric acid and 2.5 ml of Milli-Q ultrapure deionized water were added to each tube. The tubes were then returned to the Milestone ultraCLAVE digestion system for a second round of digestion at 200 °C for up to 60 min. After cooling, each sample solution was transferred to a sterile, acid-washed 50 ml polypropylene tube and diluted to 50 ml with Milli-Q ultrapure deionized water. A 1 ml aliquot of each sample was then transferred to a 15 ml pre-cleaned, metal-free polypropylene vial and diluted 10 times with Milli-Q ultrapure deionized water, resulting in a final dilution factor of 10,000.

Each prepared sample was analyzed on a triple quadrupole Agilent 8800 ICP-MS instrument. The instrument was tuned before measurement to ensure limited background interference and good signal intensity. Calibration standards of 0.1, 0.5, 1, 5, 10, and 100 ppb were prepared by diluting Inorganic Ventures primary multi-element standards and single-element standards with 2% nitric acid. Quality Control (QC) samples containing all measured elements at 0.5 and 1 ppb concentrations were also prepared. An additional 0.1 ml of indium (In) was added to each sample and used as an internal standard at a concentration of 20 ppb. The In internal standard was used to correct for instrument drift. The limit of detection of these analyses, presented in Table 2, was calculated from replicate analyses of blanks and is consistent with that reported by Cherviakouski et al.^36^. The analytical uncertainty of the measurements was calculated from replicate analyses of the CRM - JB-3 standard. The measured rock trace element composition and uncertainties are presented in Table 2.

### Fluid analysis

The pH and oxidation-reduction potential (ORP) of fluid samples were measured directly at the pilot site at the fluid sampling port using a portable multimeter. The pH electrode was regularly calibrated using Mettler Toledo pH 4.01, 7.00, and 10.01 standard buffer solutions. The uncertainty of the pH measurements was ± 0.02 based on replicate analyses of the standard buffer solutions.

Twenty-eight representative fluid samples were selected for trace element analysis. These samples were collected over a period of 11 months, spanning from before, during, and after CO_2_ injection. Each sample was filtered on site immediately after collection using a sterile 0.22 μm polyethersulfone syringe filter and collected in 50 ml CentriStar polypropylene sterile sample vials pre-rinsed with deionized water. Each fluid sample was acidified immediately after filtration by adding 2 drops of 70% ultrapure double-distilled nitric acid. The fluid samples were subsequently stored in airtight zip-lock bags and transported to the laboratory, where they were stored at 4 °C until the time of analysis.

Prior to ICP-MS analysis, each selected fluid sample was preconcentrated using an Elemental Scientific seaFAST instrument. The preconcentration method, which is based on ion exchange extraction, removes high-concentration major cations and improves the detection limit for a variety of trace elements. Approximately 10 ml of each sample was preconcentrated to yield a 0.4 ml major cation-free sample. The 0.4 ml of preconcentrated sample was then diluted to 2 ml by adding 1.5 ml Milli-Q ultrapure deionized water (18.2 MΩ.cm), and 0.1 ml of 40 ppb rhodium (Rh) as an internal standard. The Rh standard of known concentration was used to correct for instrument drift.

In addition, 3 blank solutions were prepared by adding 50 ml of Milli-Q ultrapure deionized water to sterile 50 ml acid-washed vials. The blank solutions were acidified by adding 2 drops of 70% ultrapure double-distilled nitric acid. The blank solutions were then preconcentrated and diluted to 2 ml by adding 1.5 ml of deionized water and 0.1 ml of the Rh internal standard, consistent with the measurement samples. A certified reference material (CRM) of seawater with known trace element concentrations was also preconcentrated and diluted in a similar manner and added to the measurement sequence. This CRM was used to verify the accuracy of the ICP-MS calibration.

Calibration standards for the ICP-MS analysis were prepared from an intermediate calibration solution containing 500 ppb of the target elements. One blank, and calibration standards of 1, 5, 20, and 50 ppb of the target elements, were prepared by adding appropriate amounts of the intermediate calibration solution, an internal Rh standard, and 2% ultrapure double-distilled nitric acid. Quality Control (QC) solutions containing 5 ppb of the target elements were also prepared and measured at the start, after every 10 samples, and at the end of the measurements. The corresponding volumes of the components present in the calibration standards and QC solutions are presented in Table S1. After the preconcentration of the water samples and the preparation of blanks, calibration standards, and QC solutions, the concentration of trace elements was measured on a Thermo Scientific iCAP TQ ICP-MS. The measured elements were, in order of increasing atomic number, Sc, Ti, V, Mn, Fe, Co, Ni, Cu, Zn, Ga, Y, Cd, Pb, Th, and U. The limit of detection for the measured elements, calculated from replicate blank measurements, is presented in Table S4. The analytical uncertainty was calculated from the standard deviation of replicate measurements of the CRM. The measured element concentrations and uncertainties are summarized in Table S4.

The concentration of Na in the water samples reported in Oelkers et al.^1^ and presented in Table S4 used to determine relative mobility, was measured by inductively coupled plasma optical emission spectroscopy (ICP-OES). A 1 ml aliquot of the selected fluid samples was diluted 10 times with Milli-Q ultrapure deionized water and analyzed on an Agilent 5110 ICP-OES. Calibration standards of 0.1, 1, 10, and 100 ppm were prepared from a Sigma-Aldrich major-cation ICP multi-element standard solution. The limit of detection was 0.1 ppm for Na. The analytical uncertainty was < 0.5%.

## Results

### Whole rock analyses

The whole rock compositions of the drill cuttings, measured by XRF for major elements and ICP-MS for trace elements, are presented in Tables 1 and 2. Table 1 shows the XRF composition of the basalt drill cuttings, collected from depths of 375 mbs and 665 mbs. Table 2 details the average concentrations of trace elements from 10 representative drill cutting samples taken from depths ranging from 40 to 660 m. The chemical composition of the cuttings is consistent with that of basalt, following the total alkalis and silica (TAS) chemical classification of volcanic rocks^37^. The mineral composition of drill cuttings from the Jizan Group basalt, previously reported by Oelkers et al.^1^ is presented in Table S2. The rock is primarily composed of plagioclase and pyroxene, with minor presence of amphibole, chlorite, smectite, quartz, zeolite, and calcite.

**Table 1:** The oxide composition of two drill cuttings samples collected from depths of 375 mbs and 665 mbs from the Jizan pilot production well showing major and minor composition measured by XRF.

Formula	Sample 1 (375 mbs)		Sample 2 (665 mbs)	
	Concentration (weight%)	Stat. error (%)*	Concentration (weight%)	Stat. error (%)
SiO_2_	49.0	0.1	48.5	0.1
Al_2_O_3_	13.6	0.1	16.9	0.1
Fe_2_O_3_	12.1	0.1	8.8	0.1
CaO	10.8	0.1	13.3	0.1
MgO	6.0	0.2	6.6	0.2
Na_2_O	3.2	0.5	2.4	0.6
TiO_2_	1.7	0.2	1.0	0.2
SO_3_	0.7	0.4	0.1	1.0
K_2_O	0.4	0.4	0.3	0.5
Mn_2_O_3_	0.3	1.8	0.2	2.5
LOI	2.0	-	3.4	–
Other	0.3	-	0.2	–

*The statistical error is calculated from the standard deviation of 5 repeated measurements of each sample.

**Table 2:** The average concentrations of trace elements in ten drill cuttings samples collected from depths ranging between 40–660 mbs from the Jizan pilot production well measured by ICP-MS. The concentration of trace elements in each sample is presented in Table S3.

Element	Concentration (mg/kg)	Uncertainty (%)*	Limit of detection (mg/kg)
Sc	32.93	1.51	0.03
V	274.30	0.11	0.32
Co	40.45	0.52	0.03
Ni	30.79	0.76	0.16
Cu	78.45	1.35	0.20
Zn	85.12	1.96	4.07
Ga	14.98	1.10	0.02
Y	25.57	2.16	0.01
Cd	0.18	-	-
Pb	1.62	1.28	0.04
Th	1.04	< 0.01	0.02
U	0.33	< 0.01	0.11

*The uncertainty is calculated from the standard deviation of 3 replicates of the certified reference material.

### Trace element concentration of the sampled subsurface fluids

**Fig. 2:**
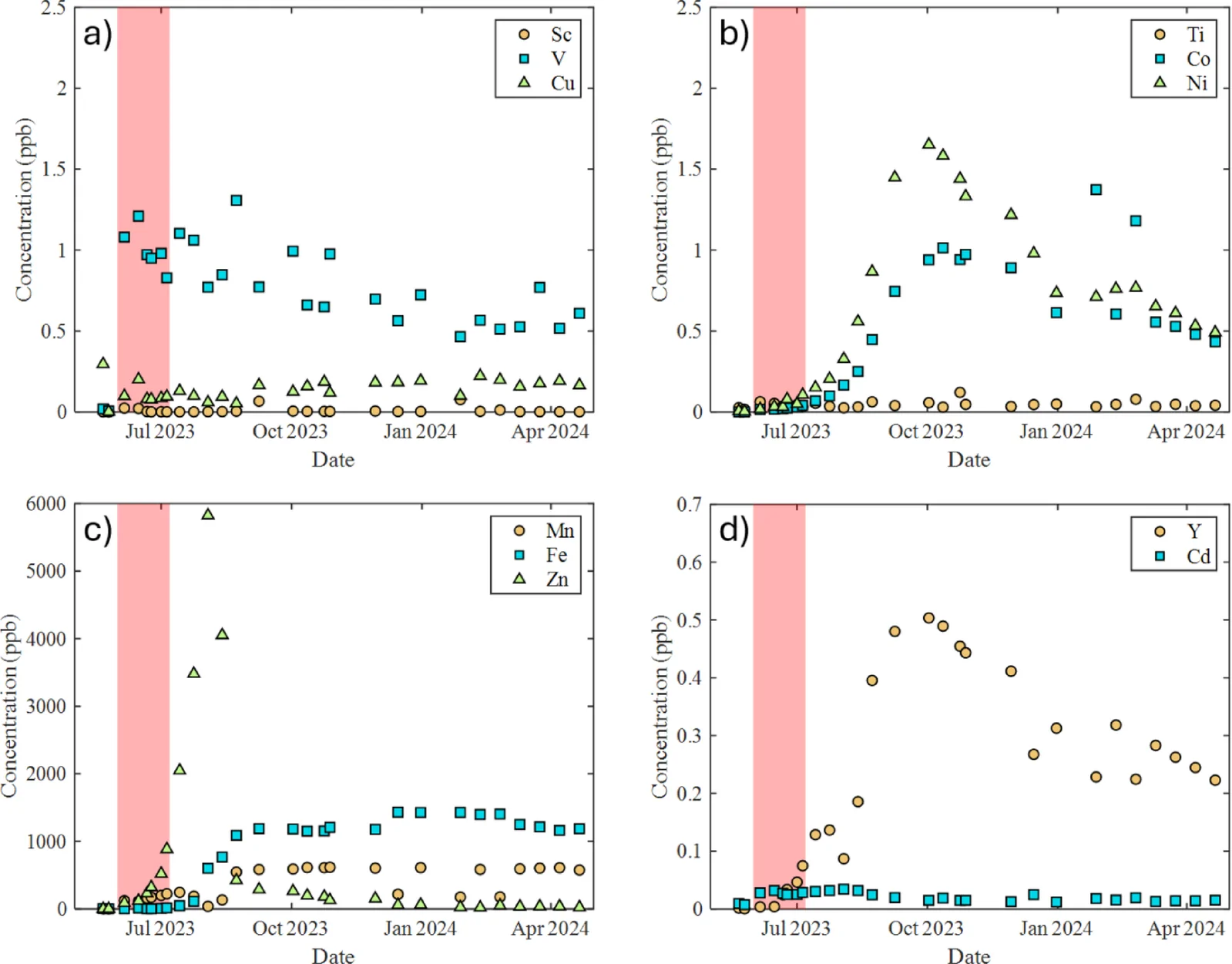
The measured concentrations of the indicated metals in the sampled reservoir fluids before, during, and after the CO_2_ injection period. The CO_2_ injection period is indicated by the semi-transparent red rectangle that overlays some of the data points. Note the different scales of the vertical axis. The uncertainties of the measurements are smaller than the symbol size for most measurements and are presented in Table S4. Note that the sharp spike in Zn concentration after CO_2_ injection in subfigure c is likely due to contamination from a galvanized steel pipe at the sampling port (see text in Discussion section).

**Fig. 3:**
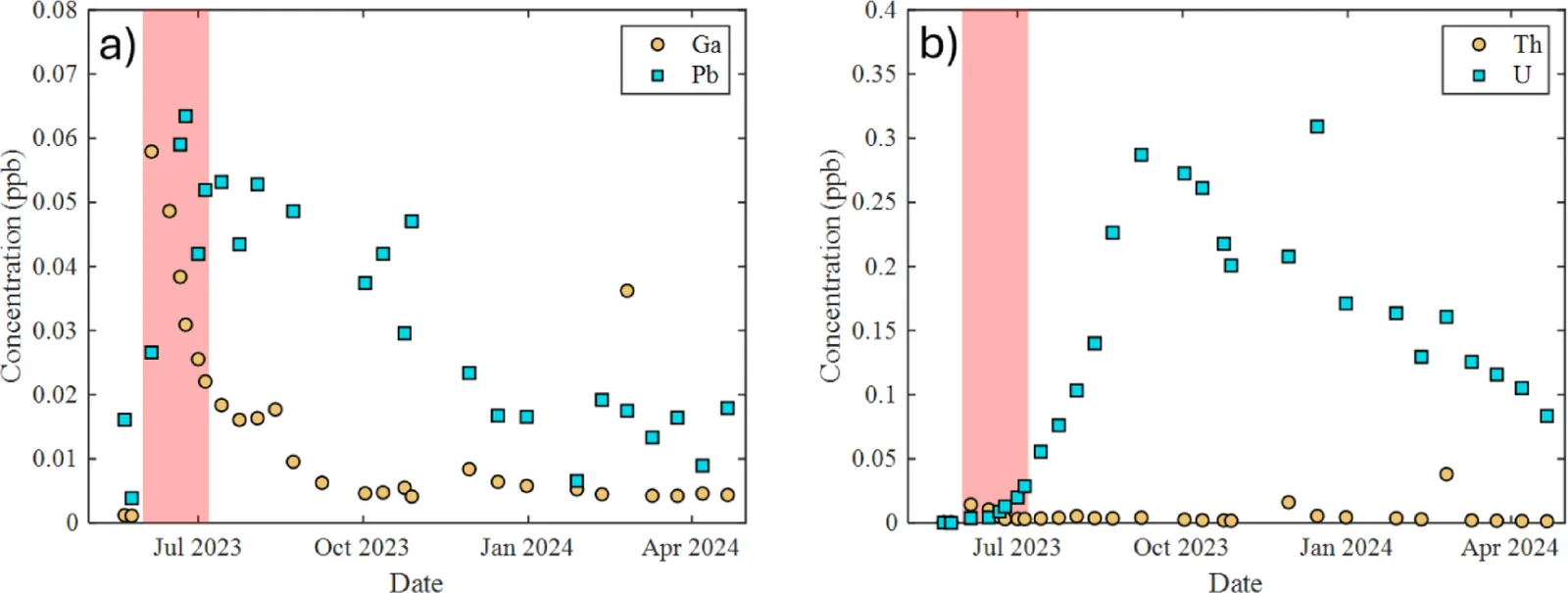
The measured concentration of post-transition metals (**a**) and actinides (**b**) in the sampled reservoir fluids before, during, and after the CO_2_ injection period. The injection period is shown by the semi-transparent red rectangle that overlays the data points. The uncertainties of the measurements are smaller than the symbol size for most measurements and are presented in Table S4.

The temporal evolution of trace element concentrations in the reservoir water samples is illustrated in Figs. 2 and 3, and listed in Table S4. The concentrations of all measured trace elements in the samples collected before the CO_2_ injection were below 1 ppb except for Fe, which ranged between 1 and 5 ppb. The concentrations of the transition metals Sc, V, and Cu, shown in Fig. 2a, remained relatively constant below 1.5 ppb during and after the CO_2_ injection period. The concentration of Ti, shown in Fig. 2b, also remained relatively constant during and after the injection period. The concentrations of Co and Ni, also shown in Fig. 2b, increased after the CO_2_ injection, peaking a few months after the end of the injection. This was followed by a decrease in their concentration over time, with the maximum measured concentration remaining below 5 ppb. The timing of the increase in the concentrations of Co and Ni appears to be approximately concurrent with that of Mn and Fe, shown in Fig. 2c. The concentrations of Mn and Fe increased substantially during the two months after the end of the CO_2_ injection and remained relatively constant at ~ 500–600 ppb and ~ 1100–1500 ppb, respectively, thereafter. The concentration of Zn, also shown in Fig. 2c, rose sharply after CO_2_ injection to ~ 6000 ppb, followed by a drop to less than 50 ppb. The concentration of Y, shown in Fig. 2d, increased after the CO_2_ injection and then decreased after peaking at ~ 0.5 ppb, showing a similar trend as Co and Ni. The concentration of Cd, also shown in Fig. 2d, slightly increased during the injection period, followed by a slight decrease during the monitoring phase of the project. All measured Cd concentrations were below 0.1 ppb. Some measured concentrations of Sc, Ti, and Th were below their limits of detection.

The concentrations of post-transition metals in the collected fluids, shown in Fig. 3a, were below 0.1 ppb for all samples. The Ga and Pb concentrations increased during the CO_2_ injection, followed by a decrease. The concentrations of the actinides, Th and U, shown in Fig. 3b, were below 0.4 ppb in all selected fluid samples. The U concentration showed an increase after CO_2_ injection, followed by a decrease, while the Th concentration remained below the LOD in most fluid samples.

### Trace element mobility

**Fig. 4:**
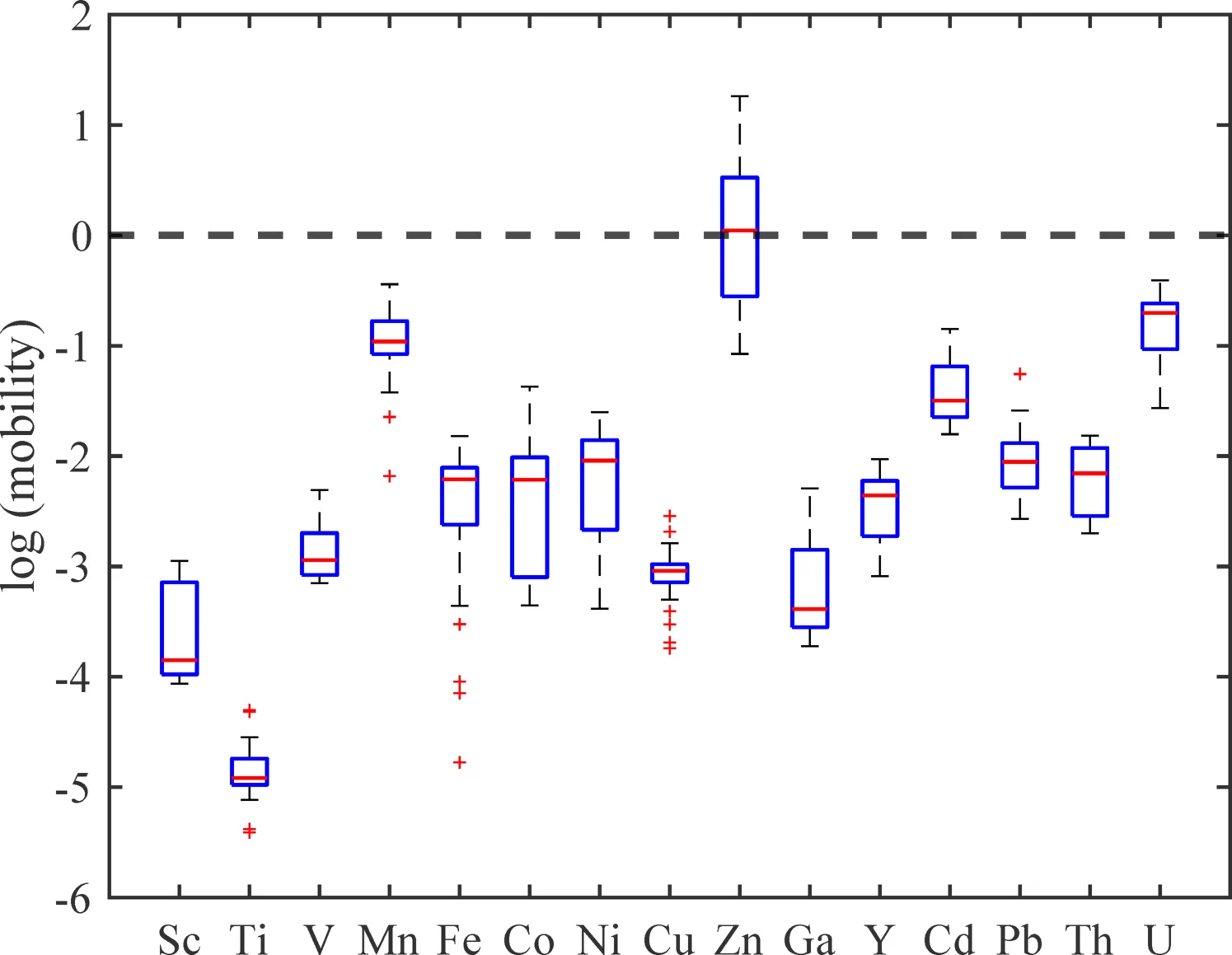
The calculated mobility of analyzed trace elements relative to Na. The black dashed line indicates an average mobility of 1 (i.e., mobility equal to that of Na). The bottom and top edges of the boxes indicate the first and third quartiles, respectively. The height of each box is the interquartile range, and the red dashes within each box represent the median. The whiskers extend to the smallest and largest values within 1.5 times the interquartile range. The red crosses indicate outliers in the data.

The relative mobility of the measured trace elements is quantified in this study by comparing the change in the sampled fluid concentrations of each element with the corresponding change in sodium concentration. Sodium was selected as a reference element for this purpose because it is a major element in the waters and rocks of the Jizan Group basalt, and as it is reported to be the most mobile major cation during basaltic rock weathering^23,38^. The relative mobility of a trace element *X* relative to Na is given by:

1 $${\mathrm{Relative}}~{\mathrm{Mobility}} = ~\frac{{\Delta X_{{water}} /\Delta Na_{{water}} }}{{X_{{rock}} /Na_{{rock}} }}$$

where Δ*X*_water_ and ΔNa_water_ represent the change in concentrations in ppb of the dissolved trace metal and Na in the water relative to their background concentrations in the Jizan water prior to CO_2_ injection, and *X*_rock_ and Na_rock_ denote the average concentrations in ppb of trace element *X* and Na in the rock. The results of this calculation are shown in Fig. 4 and Table S5. The average relative mobility of all measured trace elements remained below 20% except for Zn.

## Discussion

### Trace element mobilization

**Fig. 5:**
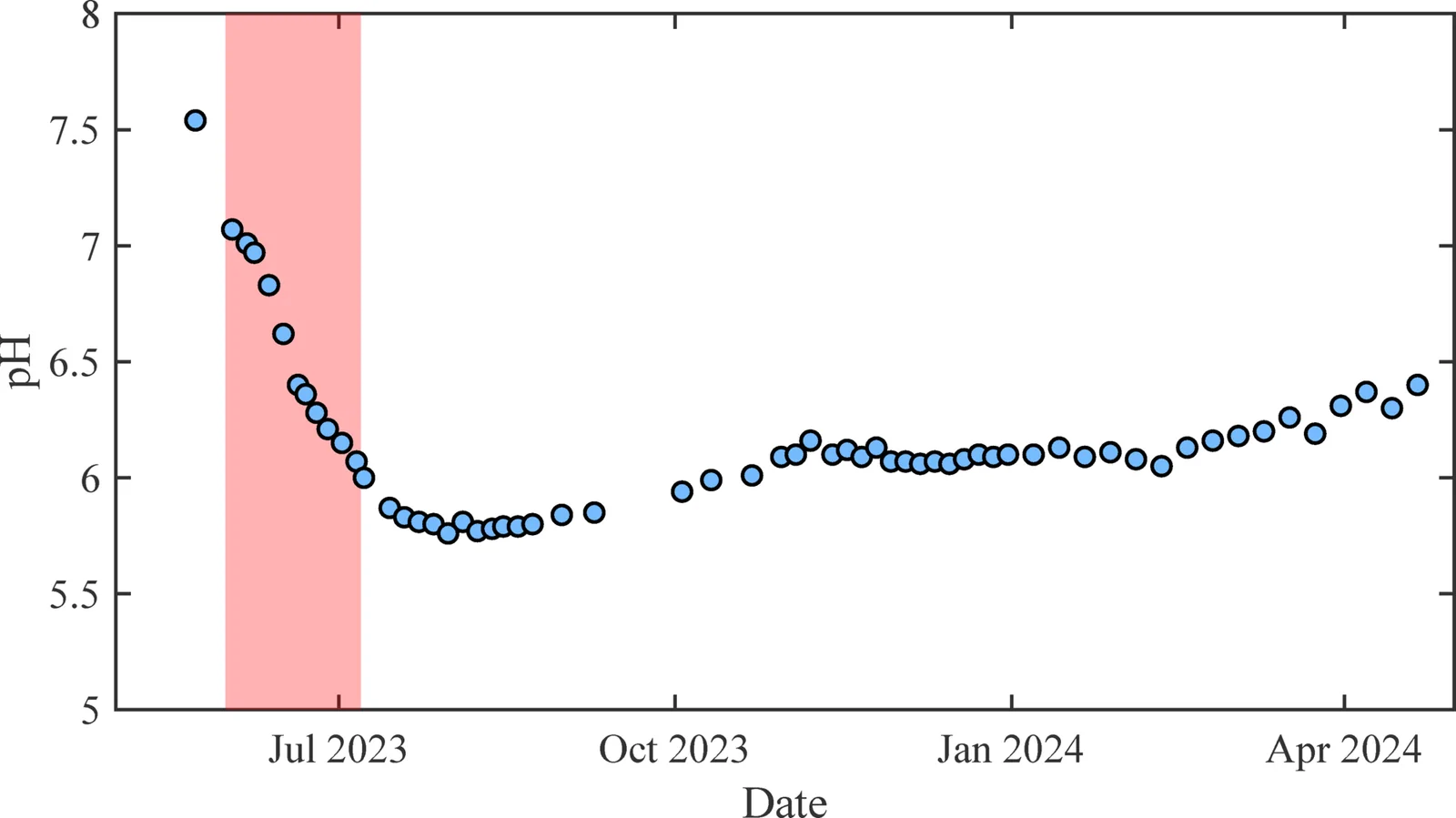
The temporal evolution of the pH (data from Oelkers et al.^1^) of the Jizan pilot production well fluids. The semi-transparent red rectangle indicates the CO_2_ injection period.

The mobilization of trace metals and potentially toxic elements presents a potential environmental risk of CO_2_ sequestration in basalt. The results presented above detail the temporal evolution of trace element concentrations in the Jizan aquifer water resulting from CO_2_-water-basalt interaction. CO_2_-basalt interaction had a negligible effect on the aqueous concentration of some elements, notably Sc, Cu, Ti, Cd, and Th. In contrast, there was a clear increase in the aqueous concentrations of V, Co, Ni, Mn, Fe, Zn, and Y in the production well fluids. The concentrations of these latter elements increased following CO_2_ injection and either plateaued or dropped with time. The increase in the concentration of trace elements in the Jizan waters can be attributed to the dissolution of basalts by the acidic CO_2_-charged injection water. The specific mineral sources of the mobilized trace elements cannot be uniquely identified from the fluid chemistry alone, because the observed aqueous concentrations likely reflect the net effect of dissolution from multiple trace element-containing mineral phases. Potential sources in the Jizan basalt include the plagioclase, pyroxene, chlorite, or minor calcite identified by XRD. The peak in most measured trace element concentrations coincides with the lowest pH measurements (see Fig. 5), consistent with the higher solubility and higher dissolution rates of major silicate and carbonate minerals at lower pH. The subsequent plateauing or drop in concentration of the released trace elements can be due to either (1) fluid mixing and dilution in the aquifer, (2) co-precipitation with secondary minerals, or (3) adsorption onto mineral surfaces.

The effect of fluid mixing in the present study is accounted for by relative mobility calculations. Specifically, if Na is not incorporated into secondary phases nor adsorbed onto mineral surfaces, and if the elements are stoichiometrically released from basalt, the calculation of a metal mobility relative to Na would account for the effect of dilution on decreasing element mobility. Sodium is chosen as a reference element as the Jizan rock is mainly composed of a Na-rich plagioclase, and plagioclase has been reported to dominate dissolution in such mafic rocks^16,39^. However, the use of Na as a reference element does not imply that all mobilized trace elements are derived from a single Na-bearing phase. Instead, Na provides a practical internal normalization for dilution and basalt reaction progress because plagioclase is abundant in the Jizan basalt and Na is expected to be largely unincorporated into precipitating secondary phases. The trace elements considered here may be released from several phases, including pyroxene, chlorite, minor carbonate phases, and accessory phases, each with potentially different trace-element amounts and dissolution rates. Despite these assumptions, the low calculated relative mobilities in Fig. 4 for most of the elements suggest that most of the trace and potentially toxic elements considered in this study are preferentially retained by solid phases in the subsurface either within leached basalt or after their release to the fluid phase by adsorption on or co-precipitation with secondary minerals.

A number of past studies concluded that the co-precipitation of trace elements with secondary minerals can lead to their relative immobilization. Flaathen et al.^18^ suggested the incorporation of Co within precipitating cobalt ferrite, and Fe into secondary ferrihydrite or oxyhydroxide phases from CO_2_-charged fluids originating from Mt. Hekla. Similarly, Marini et al.^40^ also suggested the incorporation of Co into precipitating oxyhydroxide minerals from natural groundwaters in the Bisagno valley in Genoa. These secondary minerals become more stable as the fluid pH increases over time in response to basalt dissolution. Precipitating carbonates have been reported to incorporate Sr in their structure^41,42^. Lakshtanov and Stipp^43^ and Castillo-Alvarez et al.^44^ reported the co-precipitation of Ni with calcite, forming a solid solution. Hamilton et al.^45^ observed that Fe, Mn, Cr, Ni, and Co are immobilized by incorporation into hydromagnesite during the carbonation of ultramafic mine tailings. Andersson et al.^46^ suggested the substitution of Sr, Ni, Cd, and Pb into calcite. Prieto et al.^47^ also reported the sorption of cadmium onto calcite and the formation of a carbonate solid solution. Gonzalez-Lopez et al.^48^ reported the co-precipitation of cobalt with calcite. Kohler et al.^49^ reported the uptake of Cd by aragonite. Olsson et al.^50^ reported a drop in dissolved metal concentrations, which was strongly correlated with the newly precipitated calcite travertines in a high pCO_2_ spring-fed stream in the northern slopes of the erupting Eyjafjallajökull volcano in Iceland. These observations suggest the immobilization of trace elements in groundwater systems by incorporation into secondary precipitating phases. This could explain the plateauing or decrease of trace element concentrations in the Jizan fluid samples. Both the saturation state of fluids collected from the production well during the Jizan in-situ CO_2_ mineralization pilot and the identity of precipitated minerals collected from the production well at depth suggest the precipitation of secondary carbonate minerals in the subsurface during and after the injection of CO_2_-charged water. Calcite, aragonite, and siderite were observed on the pump retrieved from the production well and these minerals were supersaturated in the sampled fluids^1^.

**Fig. 6:**
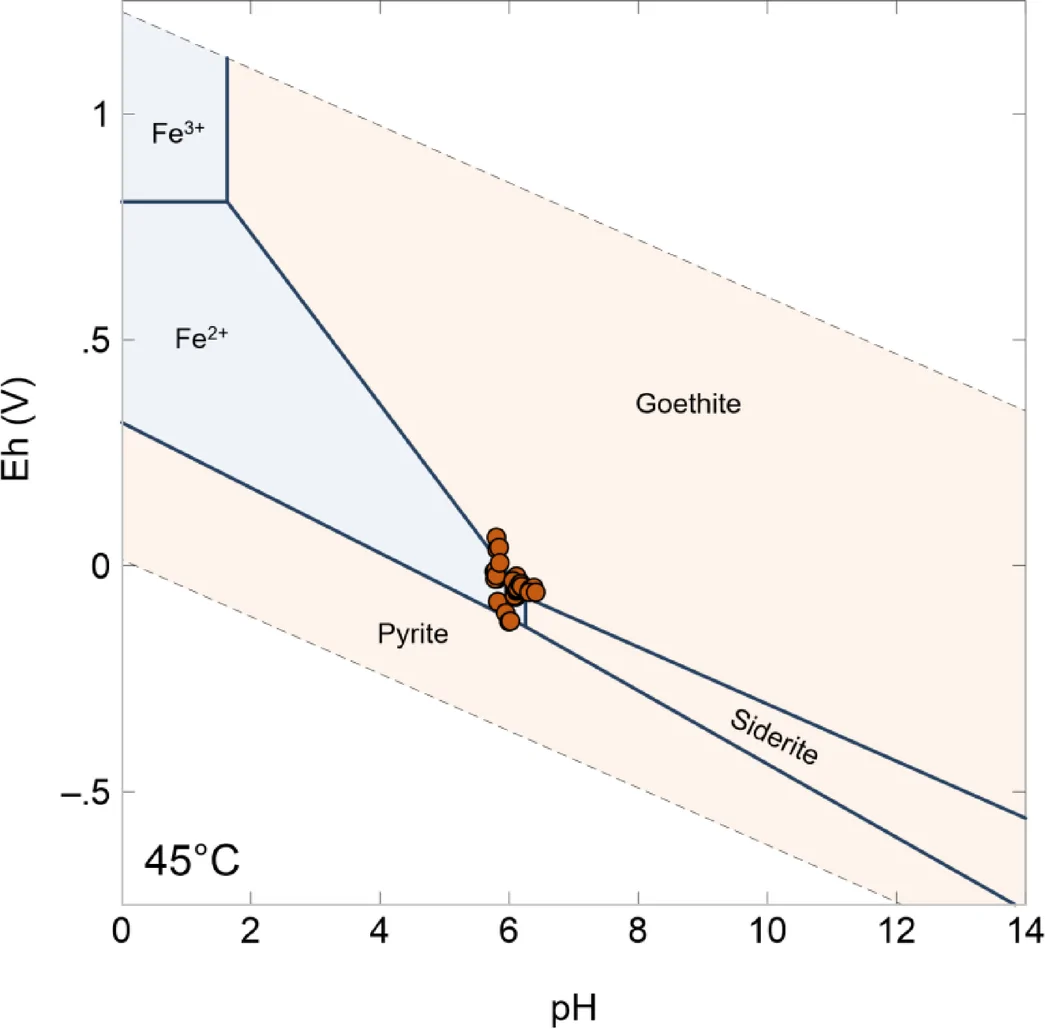
Eh-pH plot showing the Jizan pilot fluid samples’ state indicated by the orange circles. Eh-pH diagram constructed at the average temperature and Fe-C-S conditions of the Jizan fluids (45 °C, 8.5 × 10^− 06^ mol/kgw Fe, 2.0 × 10^− 02^ mol/kgw C, and 2.0 × 10^− 03^ mol/kgw S).

Trace element immobilization can also occur due to their adsorption onto mineral surfaces^18,51^. Numerous studies have reported on the adsorption of trace metals on mineral surfaces. Zachara et al.^52^ reported on the adsorption of several divalent trace metals (Ba, Sr, Cd, Mn, Zn, Co, and Ni) on calcite. Stumm^53^ presented adsorption data for the heavy metals Cd, Cr, Pb, Cu, Zn, and Ni onto the surface of ferrihydrite. Schindler and Stumm^54^ reported the adsorption of Cd, Cu, and Pb onto rutile. Hayes and Leckie^55^ reported on the adsorption of Pb onto goethite. Peacock and Sherman^56^ reported on the adsorption of Cu onto goethite and hematite. Moon and Peacock^57^ also reported on the adsorption of Cu onto ferrihydrite. The Jizan fluids are supersaturated with respect to the clay minerals montmorillonite and beidellite^1^, and are in redox equilibrium with the iron oxyhydroxide mineral goethite, as shown in Fig. 6. These high-surface-area secondary minerals provide active sites for potential adsorption of trace elements mobilized during the Jizan basalt dissolution.

The adsorption behavior of trace metals on mineral surfaces is strongly pH-dependent, and adsorption capacity of cations increases with increasing pH^58,59^. Romano et al.^23^ observed a decrease in trace element immobilization at pH > 6 during CO_2_-water-basalt interaction experiments. These observations are consistent with the trends of trace element concentrations of the dissolved metals in the Jizan water, where the measured pH of the sampled water initially drops immediately after CO_2_ injection, stabilizes, and rises during the monitoring period (see Fig. 5).

Adsorption and co-precipitation are not mutually exclusive processes. There could be an initially fast adsorption step followed by the incorporation of the adsorbed species into the crystal structure of a mineral to form a solid solution^58^. Such is observed for the adsorption of Cd on calcite surfaces^60,61^. The temporal evolution of trace element concentrations in the Jizan fluid samples suggests immobilization through co-precipitation and adsorption. The relative role of each of these processes, and the identity of the minerals that dominate the immobilization process in the Jizan subsurface, cannot be determined at present. This is due to a number of factors, including the lack of subsurface solid samples and the poor current state of quantification of the thermodynamics of the absorption/adsorption of trace elements onto mineral surfaces. Nevertheless, the observations reported in this study indicate that the combination of these processes reduces the potential risk of trace and toxic element mobility during CO_2_ disposal by mineralization in the Jizan Group basalt.

Of note is the temporal concentration of Fe in the sampled production well fluids. The Fe content of the Jizan fluids increases dramatically during August 2023 and remains elevated thereafter. The concentrations of Fe in natural waters are highly sensitive to the redox state of the fluids^62,63^. The measured redox potentials of the sampled fluids are provided in Table S4. The redox potential of the fluid samples following the increase of Fe concentration is approximately − 50 mV. At the pH of approximately 6, this redox potential is close to equilibrium with pyrite and siderite in accord with Fig. 6. It is therefore possible that the Fe in the sampled production waters after August 2023 originated from the oxidative dissolution of minor pyrite present below XRD detection limits. This process could also be involved in the formation of siderite in the subsurface. Moreover, concentrations of the elements Y, Mn, Ni, and Co also increase notably concurrently with the increase in Fe. These elements are all noted to be common trace components of natural pyrite^64^. It is important to note, however, that the concurrent increases in Fe, Mn, Co, Ni, and Y after August 2023 are consistent with release under a common set of pH-redox conditions, but they do not uniquely identify a single mineral source. In addition to possible accessory sulfides such as pyrite, these elements could be released from Fe-bearing silicates such as pyroxene and chlorite, or carbonate phases.

The observations in this study are also consistent with those of trace and potentially toxic metal mobility in response to water-dissolved CO_2_ injections during CarbFix1^17^. The concentrations of numerous metals in the fluids collected during and after the CarbFix1 injections first increased in response to the arrival of the acidic CO_2_-rich injection fluids, but returned close to their original, pre-injection values afterwards. This behavior is observed for the majority of the cations in the Jizan system. The exceptions to this are for the redox-sensitive metals Mn and Fe, which appear to remain elevated over time compared to their pre-injection values. The distinct behavior of these redox-sensitive metals is likely related to the change in the oxidation potential of the subsurface fluids during August 2023 (see Table S4).

### Implications for groundwater quality

The maximum concentrations of various trace and potentially toxic elements measured in the sampled waters are compared to the corresponding regulatory maximum acceptable levels in drinking water in Table 3. Although the injection of CO_2_-charged water into the Jizan Group basalts led to an initial increase of some trace element concentrations in the water samples, their maximum measured concentrations were significantly below the regulatory maximum acceptable levels in drinking water for all measured elements except Mn and Zn. The elevated concentration of Zn immediately after CO_2_ injection is likely due to contamination from a galvanized steel pipe connecting the wellhead to the sampling port, although dissolution of trace Zn-bearing calcite originally present in the basalt may have contributed minor Zn to these waters. Nevertheless, the fluid phase Zn concentration decreases over time following the injection through a combination of dilution and incorporation into the solid phases.

**Table 3:** The maximum measured dissolved concentrations of the analyzed elements compared to their maximum acceptable levels in drinking water based on guidelines set by various regulatory bodies. The sources for the referenced regulatory values are indicated by superscripts next to the value.

Element	Maximum measured concentration (ppb)	Acceptable levels in drinking water (ppb)
Sc	0.08	No established acceptable levels
Ti	0.12	100^65^
V	1.31	5.1^66^
Mn	617	80^67^
Fe	1431	2000^67^
Co	4.15	70^68^
Ni	1.65	70^67^
Cu	0.30	2000^67^
Zn	5824	3000^67^
Ga	0.06	18^69^
Y	0.50	No established acceptable levels
Cd	0.03	3^67^
Pb	0.11	10^67^
Th	0.04	Established levels based on radioactivity
U	0.31	30^67^

### Consequences for continuous CO_2_ injections

The results presented in this study focused on the evolution of the trace and potentially toxic elements in the waters influenced by the Jizan pilot during and after the injection of 131 tons of water-dissolved CO_2_ over a period of ~ 38 days. Various injection strategies might be considered for the upscaling of this pilot for the disposal of significantly greater CO_2_ quantities, including a continuous CO_2_-charged water injection. A question arises about the potential consequences of continuous injection on trace element mobilization. For the Jizan pilot, the injection was stopped as the partial pressure of CO_2_ gas in the recirculated subsurface fluids approached 1 bar. The measured trace element concentrations in the waters remained significantly below the maximum acceptable levels in drinking water, as these elements were immobilized by adsorption and scavenged by precipitating carbonates. This result suggests that carbonate precipitation and adsorption processes will continue to immobilize trace elements even during continuous CO_2_ injections. Such appeared to be the case during the continuous CarbFix2 injections, where the mobility of trace and potentially toxic elements appears to be limited.

During longer or larger-scale CO_2_ injections, trace-element behavior will likely be controlled by the evolving balance between element release in the acidic CO_2_-charged reaction front and subsequent immobilization during fluid neutralization, dilution, adsorption, and secondary mineral precipitation. If low-pH waters persist for longer time periods or migrate farther through the reservoir, additional mineral surfaces may dissolve, each potentially contributing trace elements and/or toxic metals to the fluid phase. Both the Jizan pilot and CarbFix observations indicate, however, that trace element concentrations in the fluids decreased or plateaued as pH increased and carbonate minerals became saturated or supersaturated.

Nevertheless, predicting the behavior of trace and potentially toxic elements during longer-duration or larger-scale carbon storage efforts remains challenging due to several uncertainties. First, the degree to which trace elements are released stoichiometrically during mineral dissolution is largely unquantified. The relative reaction rates of individual minerals in a basalt are also uncertain and depend on fluid composition. In addition, the trace-element inventory of each mineral phase in basalt remains poorly constrained. Identifying the specific source mineral phases for mobilized trace elements, and quantifying their trace-element contents and relative reactivities, would therefore substantially improve predictions of potential toxic-element mobilization during future CO_2_ injection operations. Perhaps most significantly, the thermodynamics of trace element adsorption and co-precipitation remain poorly defined, making the long-term mobility of these elements difficult to assess in detail. In addition, the degree to which a trace element is scavenged by secondary phases depends on the precipitation rates and trace element uptake capacities of these phases, which are also not well constrained. Despite these uncertainties, the results presented in this study suggest that risks associated with trace element and toxic metal mobility are greatest in low-pH fluids with elevated dissolved CO_2_ concentrations. These risks decrease substantially as pH increases and dissolved CO_2_ concentrations decrease as carbon mineralizes.

## Conclusions

The results presented in this study highlight the fate of trace and potentially toxic metals during the interaction of CO_2_-charged water with Jizan Group basalt. We measured the trace metal concentrations in the rock and in fluid samples collected before, during, and after CO_2_ injection. The key findings support the environmental viability of carbon mineralization as a permanent CO₂ sequestration strategy, as summarized below:

- Limited trace metal mobility was observed as the CO_2_-charged water interacts with the Jizan Group basalt. Despite a short transient mobilization of some trace elements, they were immobilized as the fluid phase was neutralized by dissolving basalt and the in-situ pH increased.
- The average mobility of most trace elements remained below 20% of Na mobility. Their concentrations were further decreased over time by dilution and as they were incorporated into precipitating secondary phases and adsorbed on mineral surfaces.
- The maximum measured concentrations of most trace elements remained significantly below the acceptable levels in drinking water determined by various regulatory bodies. This outcome indicates that the risk of groundwater contamination from in-situ CO_2_ mineralization in basalt formations, such as the Jizan Group, is minimal under controlled injection scenarios.

## Supplementary Information

Below is the link to the electronic supplementary material.

Supplementary Material 1

## Data Availability

All data related to this study are presented within the manuscript and an attached supplementary data file.
