# Supplementary material for: Trace element mobility during the injection of water-dissolved CO2 into basalts
Source: Sci Rep. 2026 Jun 10;16:26412. doi: 10.1038/s41598-026-57307-6 (PMC13500461; doi:10.1038/s41598-026-57307-6)
Supplement: Supplementary file 1 [file 41598_2026_57307_MOESM1_ESM.docx]

**Trace Element Mobility During the Injection of Water-Dissolved CO_2_ into Basalts**

Abdirizak Omar^1*^, Mouadh Addassi^1^, Niccolo Menegoni^1,3^, Serguey Arkadakskiy^2^, Jakub Fedorik^2^, Zeyad Ahmed^2^, Noushad Kunnummal^2^, Thomas Finkbeiner^1^, Sigurdur R. Gislason^4^, Abdulkader Afifi^1^, Hussein Hoteit^1^, Eric H. Oelkers^1,4^

^1^ Physical Science and Engineering Division, King Abdullah University of Science and Technology, Thuwal, Saudi Arabia

^2^ Saudi Aramco, Dhahran, Saudi Arabia

^3^ University of Pavia, Pavia, Italy

^4^ University of Iceland, Reykjavik, Iceland

^*^ Corresponding Author: Abdirizak Omar (abdirizakali.omar@kaust.edu.sa)

**Supplementary Data**

**Table S1.** The volumes of the components in the calibration standards and quality control solutions used in the ICP-MS measurement of the water samples.

| Solution | Concentration (µg/L) | *V*_2% HNO3_ (ml) | *V*_internal standard_ (ml) | *V*_intermediate standard_ (ml) | *V*_solution_ (ml) |
| --- | --- | --- | --- | --- | --- |
| Blank Standard | 0 | 47.5 | 2.5 | 0.0 | 50 |
| Standard 1 | 1 | 47.4 | 2.5 | 0.1 | 50 |
| Standard 2 | 5 | 47.0 | 2.5 | 0.5 | 50 |
| Standard 3 | 20 | 45.5 | 2.5 | 2.0 | 50 |
| Standard 4 | 50 | 42.5 | 2.5 | 5.0 | 50 |
| QC | 5 | 47.0 | 2.5 | 0.5 | 50 |

*Internal standard – 40 ppb Rh solution; Intermediate standard – 500 ppb multi-elements standard solution.*

**Table S2.** The mineral compositions of drill cutting samples from the injection well, collected from depths ranging between 225 and 400 mbs, determined by XRD, as previously reported by Oelkers et al. ^1^. The reported values are the average and standard deviation of 10 samples.

| Phase | Relative Abundance (weight %) | Standard Deviation |
| --- | --- | --- |
| Plagioclase (Andesine) | 36.3 | 12.2 |
| Pyroxene (Augite) | 25.7 | 5.6 |
| Amphibole (Richterite) | 2.6 | 2.5 |
| Chlorite (Clinochlore) | 11.6 | 4.06 |
| Smectite | 6.1 | 2.5 |
| Quartz | 5.3 | 2.6 |
| Zeolite (Laumontite) | 11.3 | 12.4 |
| Calcite | 1.0 | 1.9 |

**Table S3.** The concentration of trace elements in ten drilling cutting samples collected from depths ranging between 40 – 660 m from the Jizan pilot production well measured by ICP-MS.

| Sample Number | Sample Depth (m) | Sc (mg/kg) | V (mg/kg) | Co (mg/kg) | Ni (mg/kg) | Cu (mg/kg) | Zn (mg/kg) | Ga (mg/kg) | Y (mg/kg) | Cd (mg/kg) | Pb (mg/kg) | Th (mg/kg) | U (mg/kg) |
| --- | --- | --- | --- | --- | --- | --- | --- | --- | --- | --- | --- | --- | --- |
| 1 | 40 - 50 | 42.29 | 336.54 | 55.40 | 32.84 | 89.07 | 99.95 | 16.84 | 30.70 | 0.20 | 1.04 | 0.94 | 0.23 |
| 2 | 150 - 155 | 29.48 | 234.05 | 42.59 | 31.89 | 110.20 | 65.89 | 12.57 | 27.50 | 0.18 | 2.05 | 0.97 | 0.34 |
| 3 | 250 - 255 | 7.01 | 65.83 | 13.81 | 15.12 | 23.56 | 43.00 | 6.20 | 10.25 | 0.13 | 3.20 | 1.58 | 0.91 |
| 4 | 320 - 325 | 28.88 | 240.13 | 38.39 | 21.39 | 64.19 | 95.26 | 13.08 | 24.23 | 0.16 | 3.04 | 0.88 | 0.24 |
| 5 | 380 - 385 | 38.92 | 308.53 | 44.23 | 36.57 | 103.98 | 89.82 | 16.26 | 25.87 | 0.23 | 1.71 | 1.07 | 0.26 |
| 6 | 405 - 410 | 36.95 | 337.80 | 43.61 | 30.79 | 68.49 | 92.98 | 17.47 | 34.80 | 0.19 | 1.20 | 1.21 | 0.33 |
| 7 | 460 - 465 | 36.74 | 340.99 | 43.94 | 23.15 | 88.85 | 106.35 | 18.12 | 30.85 | 0.26 | 1.26 | 1.42 | 0.40 |
| 8 | 545 - 550 | 38.14 | 305.25 | 40.80 | 37.19 | 71.88 | 86.40 | 15.97 | 25.03 | 0.16 | 0.84 | 0.84 | 0.21 |
| 9 | 610 - 615 | 30.95 | 229.76 | 36.95 | 49.51 | 79.39 | 66.51 | 15.36 | 17.95 | 0.12 | 0.84 | 0.60 | 0.15 |
| 10 | 650 - 660 | 39.93 | 344.18 | 44.79 | 29.46 | 84.89 | 105.02 | 17.90 | 28.50 | 0.19 | 0.98 | 0.93 | 0.25 |
|  |  |  |  |  |  |  |  |  |  |  |  |  |  |
|  | Average | 32.93 | 274.30 | 40.45 | 30.79 | 78.45 | 85.12 | 14.98 | 25.57 | 0.18 | 1.62 | 1.04 | 0.33 |
|  | Uncertainty (%) | 1.50 | 0.10 | 0.52 | 0.76 | 1.35 | 1.96 | 1.10 | 2.16 | - | 1.28 | <0.01 | <0.01 |
|  | LOD (mg/kg) | 0.032 | 0.317 | 0.030 | 0.163 | 0.198 | 4.068 | 0.023 | 0.013 | - | 0.039 | 0.018 | 0.112 |

**Table S4.** The measured element concentrations (in ppb) of the selected samples and their 95% confidence interval uncertainties. The uncertainty is calculated from replicate analyses of the certified reference material. The limit of detection (LOD) is calculated to be equal to three times the standard deviation of the measured blank concentration. Note that the concentration of Na and Si was measured by ICP-OES and the units of the measurement are in ppm. Measurements below the limit of detection are indicated by a strikethrough. The last column on the right provides the measured ORP value of the sample in millivolts.

| Date | Sc | Ti | V | Mn | Fe | Co | Ni | Cu | Zn | Ga | Y | Cd | Pb | Th | U | Na | ORP |
| --- | --- | --- | --- | --- | --- | --- | --- | --- | --- | --- | --- | --- | --- | --- | --- | --- | --- |
| 21-May-23 | ~~0.00~~ | ~~0.03~~ | 0.02 | 0.1 | 5.1 | ~~0.00~~ | 0.01 | 0.30 | 0.3 | ~~0.00~~ | ~~0.00~~ | 0.01 | 0.02 | ~~0.00~~ | ~~0.00~~ | 622.9 | -88 |
| 25-May-23 | ~~0.00~~ | ~~0.02~~ | 0.01 | 0.0 | 1.7 | ~~0.00~~ | ~~0.00~~ | ~~0.00~~ | 0.1 | ~~0.00~~ | ~~0.00~~ | 0.01 | ~~0.00~~ | ~~0.00~~ | ~~0.00~~ | 630.0 | - |
| 5-Jun-23 | ~~0.02~~ | 0.06 | 1.08 | 122.4 | 1.4 | 0.02 | 0.02 | 0.10 | 88.5 | 0.06 | 0.00 | 0.03 | 0.03 | 0.01 | ~~0.00~~ | 639.1 | -257 |
| 15-Jun-23 | ~~0.02~~ | 0.05 | 1.21 | 113.4 | 11.1 | 0.02 | 0.03 | 0.20 | 120.0 | 0.05 | 0.00 | 0.03 | 0.10 | 0.01 | ~~0.00~~ | 641.3 | -225 |
| 21-Jun-23 | ~~0.00~~ | ~~0.04~~ | 0.97 | 158.8 | 3.1 | 0.02 | 0.03 | 0.08 | 229.2 | 0.04 | 0.02 | 0.03 | 0.06 | ~~0.00~~ | 0.01 | 642.6 | -472 |
| 24-Jun-23 | ~~0.00~~ | ~~0.04~~ | 0.95 | 170.4 | 1.4 | 0.02 | 0.08 | 0.08 | 319.7 | 0.03 | 0.03 | 0.02 | 0.06 | ~~0.00~~ | 0.01 | 645.9 | -511 |
| 1-Jul-23 | ~~0.00~~ | 0.05 | 0.98 | 199.7 | 6.7 | 0.03 | 0.05 | 0.09 | 521.1 | 0.03 | 0.05 | 0.03 | 0.04 | ~~0.00~~ | 0.02 | 649.4 | -513 |
| 5-Jul-23 | ~~0.00~~ | ~~0.03~~ | 0.83 | 223.7 | 13.4 | 0.04 | 0.11 | 0.10 | 884.0 | 0.02 | 0.07 | 0.03 | 0.05 | ~~0.00~~ | 0.03 | 655.5 | -512 |
| 14-Jul-23 | ~~0.00~~ | 0.05 | 1.10 | 245.2 | 46.5 | 0.07 | 0.15 | 0.13 | 2048.2 | 0.02 | 0.13 | 0.03 | 0.05 | ~~0.00~~ | 0.06 | 661.3 | -469 |
| 24-Jul-23 | ~~0.00~~ | ~~0.04~~ | 1.06 | 187.5 | 111.3 | 0.10 | 0.21 | 0.10 | 3479.5 | 0.02 | 0.14 | 0.03 | 0.04 | ~~0.00~~ | 0.08 | 690.0 | - |
| 3-Aug-23 | ~~0.00~~ | ~~0.03~~ | 0.77 | 37.8 | 601.2 | 0.17 | 0.33 | 0.06 | 5824.2 | 0.02 | 0.09 | 0.03 | 0.05 | 0.01 | 0.10 | 700.3 | -28 |
| 13-Aug-23 | ~~0.00~~ | ~~0.03~~ | 0.85 | 132.5 | 768.1 | 0.25 | 0.56 | 0.09 | 4051.3 | 0.02 | 0.19 | 0.03 | 0.09 | ~~0.00~~ | 0.14 | 702.1 | - |
| 23-Aug-23 | ~~0.01~~ | 0.06 | 1.31 | 545.4 | 1087.5 | 0.45 | 0.87 | 0.05 | 424.2 | 0.01 | 0.40 | 0.02 | 0.05 | ~~0.00~~ | 0.23 | 695.5 | -35 |
| 8-Sep-23 | 0.07 | ~~0.04~~ | 0.77 | 584.0 | 1187.9 | 0.75 | 1.45 | 0.17 | 287.6 | 0.01 | 0.48 | 0.02 | 0.11 | ~~0.00~~ | 0.29 | 685.7 | 5 |
| 2-Oct-23 | ~~0.01~~ | 0.06 | 0.99 | 590.5 | 1181.0 | 0.94 | 1.65 | 0.13 | 262.3 | 0.00 | 0.50 | 0.02 | 0.04 | ~~0.00~~ | 0.27 | 691.2 | -106 |
| 12-Oct-23 | ~~0.00~~ | ~~0.03~~ | 0.66 | 613.4 | 1149.6 | 1.01 | 1.58 | 0.16 | 197.4 | 0.00 | 0.49 | 0.02 | 0.04 | ~~0.00~~ | 0.26 | 665.0 | -125 |
| 24-Oct-23 | ~~0.00~~ | 0.12 | 0.65 | 611.0 | 1153.2 | 0.94 | 1.44 | 0.19 | 181.1 | 0.01 | 0.45 | 0.01 | 0.03 | ~~0.00~~ | 0.22 | 670.0 | - |
| 28-Oct-23 | ~~0.00~~ | 0.05 | 0.98 | 617.0 | 1206.8 | 0.97 | 1.33 | 0.12 | 128.7 | 0.00 | 0.44 | 0.01 | 0.05 | ~~0.00~~ | 0.20 | 666.0 | - |
| 29-Nov-23 | ~~0.01~~ | ~~0.03~~ | 0.70 | 604.2 | 1176.6 | 0.89 | 1.22 | 0.18 | 152.7 | 0.01 | 0.41 | 0.01 | 0.02 | 0.02 | 0.21 | 679.8 | -60 |
| 15-Dec-23 | ~~0.00~~ | 0.05 | 0.56 | 217.1 | 1431.3 | 4.15 | 0.98 | 0.18 | 57.2 | 0.01 | 0.27 | 0.02 | 0.02 | 0.01 | 0.31 | 672.6 | -33 |
| 31-Dec-23 | ~~0.00~~ | 0.05 | 0.72 | 611.3 | 1427.1 | 0.61 | 0.74 | 0.19 | 62.9 | 0.01 | 0.31 | 0.01 | 0.02 | ~~0.00~~ | 0.17 | 672.5 | -56 |
| 28-Jan-24 | 0.08 | ~~0.03~~ | 0.47 | 174.6 | 1427.9 | 1.37 | 0.71 | 0.10 | 25.5 | 0.01 | 0.23 | 0.02 | 0.01 | ~~0.00~~ | 0.16 | 667.6 | -51 |
| 11-Feb-24 | ~~0.00~~ | 0.05 | 0.57 | 584.7 | 1397.7 | 0.61 | 0.76 | 0.22 | 20.7 | 0.00 | 0.32 | 0.02 | 0.02 | ~~0.00~~ | 0.13 | 681.5 | -35 |
| 25-Feb-24 | ~~0.01~~ | 0.08 | 0.51 | 176.7 | 1403.6 | 1.18 | 0.77 | 0.20 | 49.3 | 0.04 | 0.22 | 0.02 | 0.02 | 0.04 | 0.16 | 674.8 | -42 |
| 10-Mar-24 | ~~0.00~~ | ~~0.03~~ | 0.53 | 593.7 | 1250.2 | 0.56 | 0.65 | 0.16 | 33.5 | 0.00 | 0.28 | 0.01 | 0.01 | ~~0.00~~ | 0.13 | 671.0 | -50 |
| 24-Mar-24 | ~~0.00~~ | 0.05 | 0.77 | 603.8 | 1214.2 | 0.53 | 0.61 | 0.18 | 37.5 | 0.00 | 0.26 | 0.01 | 0.02 | ~~0.00~~ | 0.12 | 654.2 | -46 |
| 7-Apr-24 | ~~0.00~~ | ~~0.04~~ | 0.52 | 609.7 | 1161.9 | 0.48 | 0.53 | 0.19 | 38.0 | 0.00 | 0.24 | 0.01 | 0.01 | ~~0.00~~ | 0.11 | 656.9 | -50 |
| 21-Apr-24 | ~~0.00~~ | ~~0.04~~ | 0.61 | 575.5 | 1186.0 | 0.43 | 0.49 | 0.17 | 23.5 | 0.00 | 0.22 | 0.02 | 0.02 | ~~0.00~~ | 0.08 | 644.5 | -60 |
|  |  |  |  |  |  |  |  |  |  |  |  |  |  |  |  |  |  |
| Uncertainty (%) | 26.71 | 6.77 | 2.31 | 2.0 | 51.5 | 2.98 | 2.93 | 3.22 | 1.07 | 0.587 | 0.349 | 1.844 | 2.310 | 6.325 | 1.249 | <1 |  |
| LOD (ppb) | 0.055 | 0.043 | 0.001 | 0.002 | 0.015 | <0.001 | 0.002 | 0.016 | 0.003 | 0.003 | <0.001 | 0.001 | 0.015 | 0.006 | <0.001 | 10 |  |

**Table S5.** The calculated mobilities of the analyzed trace elements relative to Na according to Equation (1).

| Date | Sc | Ti | V | Mn | Fe | Co | Ni | Cu | Zn | Ga | Y | Cd | Pb | Th | U |
| --- | --- | --- | --- | --- | --- | --- | --- | --- | --- | --- | --- | --- | --- | --- | --- |
| 25-May-23 | 0.000 | 0.000 | 0.000 | 0.000 | 0.000 | 0.000 | 0.001 | 0.000 | 0.007 | 0.000 | 0.000 | 0.000 | 0.036 | 0.000 | 0.000 |
| 5-Jun-23 | 0.001 | 0.000 | 0.005 | 0.102 | 0.000 | 0.001 | 0.000 | 0.002 | 1.319 | 0.005 | 0.000 | 0.141 | 0.008 | 0.012 | 0.000 |
| 15-Jun-23 | 0.001 | 0.000 | 0.005 | 0.083 | 0.000 | 0.001 | 0.001 | 0.003 | 1.576 | 0.004 | 0.000 | 0.125 | 0.055 | 0.011 | 0.000 |
| 21-Jun-23 | 0.000 | 0.000 | 0.004 | 0.109 | 0.000 | 0.001 | 0.001 | 0.001 | 2.816 | 0.003 | 0.001 | 0.116 | 0.026 | 0.000 | 0.032 |
| 24-Jun-23 | 0.000 | 0.000 | 0.003 | 0.100 | 0.000 | 0.000 | 0.002 | 0.001 | 3.365 | 0.002 | 0.001 | 0.050 | 0.022 | 0.000 | 0.027 |
| 1-Jul-23 | 0.000 | 0.000 | 0.003 | 0.102 | 0.000 | 0.001 | 0.001 | 0.001 | 4.762 | 0.002 | 0.002 | 0.086 | 0.010 | 0.000 | 0.047 |
| 5-Jul-23 | 0.000 | 0.000 | 0.002 | 0.093 | 0.000 | 0.001 | 0.002 | 0.001 | 6.569 | 0.001 | 0.002 | 0.070 | 0.012 | 0.000 | 0.058 |
| 14-Jul-23 | 0.000 | 0.000 | 0.002 | 0.086 | 0.000 | 0.001 | 0.002 | 0.001 | 12.923 | 0.001 | 0.003 | 0.060 | 0.010 | 0.000 | 0.098 |
| 24-Jul-23 | 0.000 | 0.000 | 0.001 | 0.038 | 0.000 | 0.001 | 0.002 | 0.000 | 12.564 | 0.000 | 0.002 | 0.034 | 0.004 | 0.000 | 0.075 |
| 3-Aug-23 | 0.000 | 0.000 | 0.001 | 0.007 | 0.002 | 0.001 | 0.003 | 0.000 | 18.233 | 0.000 | 0.001 | 0.030 | 0.005 | 0.003 | 0.081 |
| 13-Aug-23 | 0.000 | 0.000 | 0.001 | 0.023 | 0.003 | 0.002 | 0.005 | 0.000 | 12.394 | 0.000 | 0.002 | 0.029 | 0.011 | 0.000 | 0.110 |
| 23-Aug-23 | 0.000 | 0.000 | 0.001 | 0.102 | 0.004 | 0.003 | 0.008 | 0.000 | 1.415 | 0.000 | 0.004 | 0.016 | 0.005 | 0.000 | 0.198 |
| 8-Sep-23 | 0.001 | 0.000 | 0.001 | 0.126 | 0.005 | 0.006 | 0.015 | 0.001 | 1.109 | 0.000 | 0.006 | 0.018 | 0.018 | 0.000 | 0.289 |
| 2-Oct-23 | 0.000 | 0.000 | 0.001 | 0.117 | 0.005 | 0.007 | 0.016 | 0.001 | 0.930 | 0.000 | 0.006 | 0.017 | 0.004 | 0.000 | 0.247 |
| 12-Oct-23 | 0.000 | 0.000 | 0.001 | 0.197 | 0.008 | 0.012 | 0.025 | 0.001 | 1.134 | 0.000 | 0.009 | 0.027 | 0.006 | 0.000 | 0.386 |
| 24-Oct-23 | 0.000 | 0.000 | 0.001 | 0.176 | 0.007 | 0.010 | 0.020 | 0.001 | 0.930 | 0.000 | 0.008 | 0.000 | 0.003 | 0.000 | 0.292 |
| 28-Oct-23 | 0.000 | 0.000 | 0.002 | 0.194 | 0.008 | 0.011 | 0.021 | 0.001 | 0.722 | 0.000 | 0.008 | 0.000 | 0.009 | 0.000 | 0.290 |
| 29-Nov-23 | 0.000 | 0.000 | 0.001 | 0.144 | 0.006 | 0.008 | 0.014 | 0.001 | 0.649 | 0.000 | 0.006 | 0.000 | 0.000 | 0.007 | 0.231 |
| 15-Dec-23 | 0.000 | 0.000 | 0.001 | 0.059 | 0.008 | 0.043 | 0.013 | 0.001 | 0.277 | 0.000 | 0.004 | 0.023 | 0.000 | 0.004 | 0.390 |
| 31-Dec-23 | 0.000 | 0.000 | 0.001 | 0.167 | 0.008 | 0.006 | 0.010 | 0.001 | 0.306 | 0.000 | 0.005 | 0.000 | 0.000 | 0.000 | 0.214 |
| 28-Jan-24 | 0.001 | 0.000 | 0.001 | 0.053 | 0.009 | 0.016 | 0.010 | 0.001 | 0.137 | 0.000 | 0.004 | 0.026 | 0.003 | 0.000 | 0.224 |
| 11-Feb-24 | 0.000 | 0.000 | 0.001 | 0.135 | 0.007 | 0.005 | 0.009 | 0.001 | 0.084 | 0.000 | 0.004 | 0.020 | 0.000 | 0.000 | 0.139 |
| 25-Feb-24 | 0.000 | 0.000 | 0.001 | 0.046 | 0.007 | 0.012 | 0.010 | 0.001 | 0.229 | 0.001 | 0.003 | 0.022 | 0.000 | 0.015 | 0.193 |
| 10-Mar-24 | 0.000 | 0.000 | 0.001 | 0.167 | 0.007 | 0.006 | 0.009 | 0.001 | 0.167 | 0.000 | 0.005 | 0.000 | 0.003 | 0.000 | 0.169 |
| 24-Mar-24 | 0.000 | 0.000 | 0.002 | 0.261 | 0.011 | 0.009 | 0.013 | 0.002 | 0.288 | 0.000 | 0.007 | 0.000 | 0.000 | 0.000 | 0.240 |
| 7-Apr-24 | 0.000 | 0.000 | 0.001 | 0.243 | 0.009 | 0.007 | 0.010 | 0.001 | 0.269 | 0.000 | 0.006 | 0.000 | 0.004 | 0.000 | 0.202 |
| 21-Apr-24 | 0.000 | 0.000 | 0.002 | 0.361 | 0.015 | 0.010 | 0.015 | 0.002 | 0.260 | 0.000 | 0.008 | 0.053 | 0.000 | 0.000 | 0.231 |
| Average | 0.000 | 0.000 | 0.002 | 0.127 | 0.005 | 0.007 | 0.009 | 0.001 | 3.286 | 0.001 | 0.004 | 0.037 | 0.008 | 0.002 | 0.172 |
| log (average) | -3.85 | -4.91 | -2.78 | -0.90 | -2.30 | -2.16 | -2.04 | -3.01 | 0.52 | -3.10 | -2.39 | -1.43 | -2.12 | -2.70 | -0.77 |
